# Precision virotherapies: Coming soon

**DOI:** 10.18632/oncotarget.26280

**Published:** 2018-11-02

**Authors:** Hanni Uusi-Kerttula, Alan L. Parker

**Affiliations:** Division of Cancer and Genetics, Cardiff University School of Medicine, Cardiff CF14 4XN, United Kingdom

**Keywords:** oncolytic, adenovirus, virotherapy, αvβ6 integrin, targeting

The recent licensing of talimogene laherparepvec (Imlygic™) for treatment of malignant melanoma by the FDA and EMA provides proof of the long heralded clinical potential of oncolytic viruses (OV) [1]. Some three decades on from their first clinical evaluation, mounting evidence suggests that OV may be ready to deliver on their promise as powerful immunostimulatory anti-cancer agents.

The use of virotherapies offers unique advantages compared to conventional drug- or antibody-based anti-cancer agents. A major advantage lies in the capacity of virotherapies to self-amplify following infection of cancer cells – a feature unique to biological agents. Infected cells produce tens of thousands of daughter virions, before lysing the infected cell, spreading virions to surrounding cells, repeating and amplifying the process. The lytic nature of OV-induced cell death is highly immunogenic. It induces a robust anti-tumor immune response, which can be further exacerbated through the *in situ*, virus-mediated over-expression of engineered immunostimulatory transgenes. Virotherapies thus have the potential to turn immunologically “cold” tumors “hot”, with increasing evidence suggesting resistant tumors can be sensitized to subsequent immunotherapies through pre-treatment with OV, which results in significant tumor regression [2, 3].

**Figure 1:**
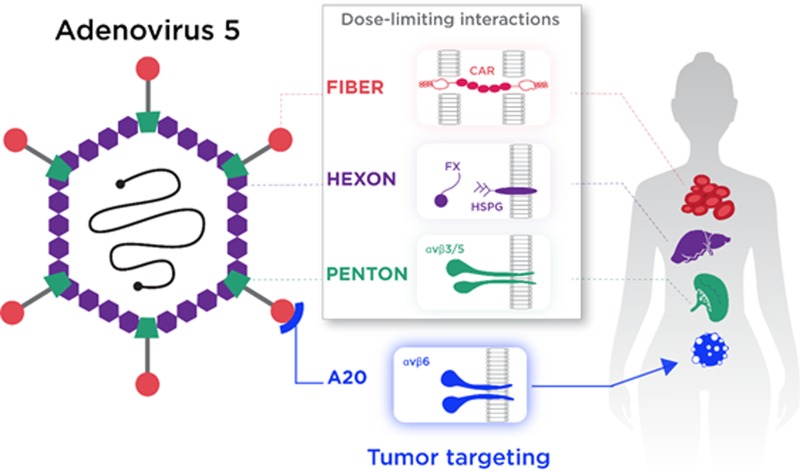
Refinement of Ad5 into a highly tumorselective virotherapy Successful intravascular tumortargeting of Ad5 is limited by interactions involving each of the major capsid proteins, resulting in “off-target” sequestration, predominantly in the liver and spleen. To overcome these limitations, the Ad5_NULL_ oncolytic virus harbours mutations in each of the major capsid proteins – hexon, penton base and fiber – to preclude uptake via all described native routes. To efficiently target the Ad5_NULL_ platform vector to transformed epithelial cells expressing αvβ6 integrin, the vector contains an additional 20-amino-acid (A20) peptide sequence. The resultant virotherapy, Ad5_NULL_-A20, selectively and efficiently infects aggressively transformed cells via αvβ6 integrin.

Unfortunately, viruses – including those based on the well-studied oncolytic vector, adenovirus serotype 5 (Ad5) – have not evolved to be intrinsically tumor-selective. Rather, they have evolved sophisticated means to infect healthy cells, efficiently delivering their DNA payload to the nucleus. As such, the selectivity of OV for cancer cell killing most commonly relies upon subtle changes or reassortments of viral early genes to allow preferential replication within transformed cells, with minimal replication in non-transformed cells [4, 5]. Using this approach, the pool of vector available for transducing transformed cells is depleted by uptake in “off-target” organs, with consequent dose limiting toxicities. Clearly, the therapeutic index of virotherapies, especially those introduced via the intravenous route, could be improved through a systemic and rational redesign of the viral capsid to preclude native means of infection. For Ad5, interactions leading to vector sequestration are relatively well defined. Cell entry is initiated by interaction between the extended fiber knob protein to the primary receptor, Coxsackie and Adenovirus Receptor (CAR) [6]. Following attachment, cellular internalization is stimulated by secondary interactions between the penton base and cellular αvβ3/5 integrins, resulting in uptake via clathrin-coated endosomes [7]. For cancer therapies, CAR and αvβ3/5 integrins represent poor targets for therapeutic delivery of biologics. CAR is expressed in most organs, but is anatomically restricted to tight junctions [8], whilst loss of CAR expression with tumor progression has been previously reported [9, 10]. The expression of CAR on human erythrocytes may also act as a “sink” for circulating Ad5, trapping virions within the bloodstream. Furthermore, binding of the Ad5 virion to the co-receptor αvβ3/5 has been reported to result in sequestration by splenic macrophages, degrading virions and inducing a potent innate antiviral response, with consequent dose-limiting toxicities [11, 12]. Of critical relevance for targeting metastases is the high affinity interaction between the Ad5 major coat protein, hexon, and circulating coagulation zymogen, FX. This high-affinity interaction results in rapid, selective and efficient uptake of Ad5 by hepatocytes via heparan sulphate proteoglycans (HSPG) [13–15]. In summary, each of the major Ad5 capsid proteins – hexon, penton base and fiber – plays a major role in “off-target” uptake of Ad5, inducing dose-limiting toxicities whilst rapidly and efficiently depleting the pool of vector available for therapeutic delivery to the tumor (Figure 1).

To generate a refined, tumor-selective Ad5 virotherapy, Uusi-Kerttula et al recently reported the construction of an oncolytic vector harboring modifications in each of the major capsid proteins to preclude all native routes of infection [16]. The resultant, triply modified platform vector Ad5_NULL_ is defective in uptake and thus cannot be produced without additional modifications to empower the vector with a new means of cellular entry, together with a surrogate rescue cell line for propagation. To address this, the authors engineered αvβ6 tropism into the vector, through incorporation of a 20-amino-acid αvβ6 peptide ligand, A20, into the Ad5 fiber knob. αvβ6 is a compelling candidate for tumor targeting: it is undetectable in healthy epithelial cells, but over-expressed in a range of aggressively transformed cancers, where it is involved in TGF-β signaling, invasion and metastasis [17], and where over-expression correlates with poor patient prognosis [18]. The resultant vector, Ad5_NULL_-A20, was rescued in 293-β6 cells, and evaluated further. *In vitro*, the authors demonstrated Ad5_NULL_-A20 was devoid of native means of cellular infection, and infected cells entirely in an αvβ6-dependent fashion. Experiments performed in the presence of neutralizing serum demonstrated that the virotherapy appeared capable of at least partially overcoming pre-existing anti-Ad5 immunity. *In vivo*, in non-tumor-bearing animals, Ad5_NULL_-A20 completely abolished “off-target” uptake via the liver and spleen, including a remarkable 7-log reduction in vector genomes recovered from the liver. In a ovarian cancer xenograft model, Ad5_NULL_-A20 vector could be visualized infecting tumor cells selectively and efficiently in animals with peritoneal tumors. The treatment resulted in complete survival in the Ad5_NULL_-A20 cohort after >100 days, as opposed to a median survival time of ~55 days for mice treated with unrefined OV, and ~45 days for untreated mice. This efficacy study also highlighted the absolute requirement for the combination of both de-targeting and re-targeting for effective treatment, since mice treated with Ad5-A20 (containing the αvβ6 re-targeting peptide insert but no de-targeting modifications) failed to improve survival over unrefined Ad5.

The production and characterization of the Ad5_NULL_ platform and efficient retargeting to αvβ6 integrin via A20 peptide represents a milestone in the development of fully refined virotherapies for personalized cancer treatment. Whilst the anti-cancer activity addressed to date has focused solely on oncolytic potential of the vector, additional refinement to express relevant immunomodulatory transgenes is certain to further enhance anticancer effects. It remains to be seen whether the Ad5_NULL_ platform can be adapted to tailor virotherapies towards other tumor-restricted receptors. Assuming this will be possible, this technology advances us towards an era where the development of precision virotherapies for individualized patient treatment may well become reality [19].
